# Seroprevalence of SARS-CoV-2 spike IgG antibodies after the second BNT162b2 mRNA vaccine in Japanese kidney transplant recipients

**DOI:** 10.1038/s41598-022-09897-0

**Published:** 2022-04-07

**Authors:** Tomoko Hamaya, Shingo Hatakeyama, Tohru Yoneyama, Yuki Tobisawa, Hirotake Kodama, Takeshi Fujita, Reiichi Murakami, Naoki Fujita, Teppei Okamoto, Hayato Yamamoto, Takahiro Yoneyama, Yasuhiro Hashimoto, Hisao Saitoh, Shunji Narumi, Hirofumi Tomita, Chikara Ohyama

**Affiliations:** 1 Department of Urology, Hirosaki University School of Medicine, 5 Zaifu-cho, Hirosaki, Aomori 036-8562 Japan; 2 Department of Advanced Blood Purification Therapy, Hirosaki University School of Medicine, 5 Zaifu-cho, Hirosaki, Aomori 036-8562 Japan; 3 Department of Glycotechnology, Center for Advanced Medical Research, Hirosaki University School of Medicine, 5 Zaifu-cho, Hirosaki, Aomori 036-8562 Japan; 4 Department of Cardiology and Nephrology, Hirosaki University School of Medicine, 5 Zaifu-cho, Hirosaki, Aomori 036-8562 Japan; 5 Department of Advanced Transplant and Regenerative Medicine, Hirosaki University School of Medicine, 5 Zaifu-cho, Hirosaki, Aomori 036-8562 Japan; 6 Department of Urology, Oyokyo Kidney Research Institute, 90 Kozawayamazaki, Hirosaki, Aomori 036-8243 Japan; 7 Department of Transplant Nephrology and Surgery, Japanese Red Cross Aichi Medical Center Nagoya Daini Hospital, Nagoya, Japan

**Keywords:** Transplant immunology, Renal replacement therapy, Infectious diseases, Viral infection

## Abstract

We aimed to evaluate the seroprevalence and investigated factors associated with seropositivity after the second SARS-CoV-2 mRNA vaccination in kidney transplant (KT) recipients. This retrospective study conducted between June and November 2021 included 106 KT recipients and 127 healthy controls who received the second dose of the BNT162b2 mRNA vaccine at least 7 days before the measurement of antibody titers. The antibody titer against the receptor-binding domain of SARS-CoV-2 spike (S) protein was determined. We compared seroprevalence rates (immunoglobulin G [IgG] level of ≥ 0.8 or ≥ 15 U/mL) between the healthy controls and KT recipients and identified factors associated with impaired humoral response. The seroprevalence rate of the healthy controls and KT recipients was 98% and 22%, respectively. Univariate logistic regression analysis revealed that age > 53 years, rituximab use, mycophenolate mofetil use, and KT vintage < 7 years were negatively associated with the rate of anti-SARS-CoV-2 S IgG ≥ 15 U/mL in KT recipients. ABO blood type incompatible KT was not significantly associated with seroprevalence. Humoral response after the second BNT162b2 mRNA vaccine was greatly hindered by immunosuppression therapy in KT recipients. Older age, rituximab use, mycophenolate mofetil use, and KT vintage may play key roles in seroconversion.

## Introduction

Severe acute respiratory syndrome coronavirus 2 (SARS-CoV-2) infection is a critical disease associated with high mortality rate in kidney transplant (KT) recipients with immunosuppression^1^, for whom SARS-CoV-2 vaccination is recommended for infection prevention. However, several studies reported that KT recipients exhibited a significantly impaired response to standard dose of SARS-CoV-2 mRNA-based vaccination compared to the general population^2–7^. Sufficient data are not available for KT recipients, who were not included in SARS-CoV-2 vaccine clinical trials^8^. Additionally, most studies evaluating immunoglobulin G (IgG) antibody titer against SARS-CoV-2 mRNA vaccines (Pfizer/BioNTech BNT162b2 or Moderna mRNA-1273) in KT recipients were from Western countries^2–7^. As KT protocols vary across countries and regions, the vaccine efficacy has not been fully validated in KT recipients in Japan. In Japan, ABO blood-type incompatible (ABOi) KT protocols with strong immunosuppression strategies are necessary due to the absence of donor exchange programs and the serious donor shortage^9–13^. Currently, one-third of the recipients undergo ABOi KT with rituximab desensitization. However, the anti-SARS-CoV-2 IgG seroconversion rate after the second SARS-CoV-2 mRNA-based vaccination in patients who undergo ABOi KT with contemporary immunosuppressive strategies remains unknown. Therefore, we measured the titers of IgG antibodies directed against the receptor-binding domain of SARS-CoV-2 spike (S) protein and investigated risk factors for inadequate humoral response after the second dose of the Pfizer/BioNTech BNT162b2 mRNA vaccine in KT recipients, including those who underwent ABOi KT.

## Results

The background characteristics of the study cohort are summarized in Table 1. Briefly, the median ages were 68 (IQR: 38–77) and 56 (IQR: 44–65) years in the controls and KT recipients, respectively. Rituximab was administrated in 43 (41%) KT recipients, including 24 (23%) ABOi KT recipients and 19 (18%) ABOc KT recipients. Biopsy-proven rejection and viral infections before enrollment in the current study were observed in 10 (9%) and 11 (10%) patients, respectively. Steroids were used in most of all recipients (n = 97, 92%), with a median prednisone dose of 5.0 mg. All recipients received combined immunosuppressive therapy including a median of three agents. Everolimus was used in 12 recipients. The median period after KT was 6.3 years. No recipient experienced biopsy-proven rejection or viral events during the current study period.

**Table 1:** Background of participants.

	Ctrl	Recipients			
		All	Antibody titer	Antibody titer	P value
			< 15 U/mL	≥ 15 U/mL	(< 15 vs. ≥ 15 U/mL)
n	127	106	83	23	
Age, years	68 (38–77)	56 (44–65)	59 (46–66)	44 (38–55)	< 0.001
Male, n (%)	91 (72%)	64 (60%)	51 (61%)	13 (57%)	0.81
Antibody titer ≥ 15 U/mL, n	125 (98%)				
Antibody titer 0.40–15 U/mL, n (%)	127 (100%)	11 (10%)	11 (13%)	0 (0%)	0.196
Months after 1st-vaccine	3.5 (1.8–5.8)	1.9 (1.4–2.4)			
Age at KT, years		46 (34–58)	49 (38–60)	34 (27–42)	< 0.001
Median KT vintage, years		6 (3–12)	5 (3–9)	12 (7–15)	< 0.001
Median dialysis vintage, years		1 (0.3–3)	1 (0.1–3)	2 (0.3–3)	< 0.001
Cadaver KT, n (%)		14 (13%)	11 (13%)	3 (13%)	1.000
**Primary kidney disease, n (%)**					
Glomerular		51 (48%)	41 (49%)	10 (43%)	0.645
Vascular		4 (3.8%)	4 (4.8%)	0 (0.0%)	0.575
Interstitial		3 (2.8%)	1 (1.2%)	2 (8.7%)	0.118
Polycystic kidney disease		8 (7.5%)	5 (6.0%)	3 (13%)	0.367
Diabetes		14 (13%)	13 (16%)	1 (4.3%)	0.294
Others		26 (25%)	19 (23%)	7 (30%)	0.584
ABO blood type incompatible KT, n (%)		24 (23%)	22 (27%)	2 (8.7%)	0.093
**Immunosuppressant regimen, n (%)**					
Tacrolimus		90 (85%)	73 (88%)	17 (74%)	0.109
Cyclosporine		12 (11%)	8 (10%)	4 (17%)	0.287
Mycophenolate mofetil		89 (84%)	76 (92%)	13 (57%)	< 0.001
Azathioprine		9 (8.5%)	3 (3.6%)	6 (26%)	0.003
Everolimus		12 (11%)	8 (9.6%)	4 (17%)	0.287
Steroids		97 (92%)	76 (92%)	21 (91%)	1.000
Rituximab		43 (41%)	38 (46%)	5 (22%)	0.054
Any history of rejection events, n (%)		10 (9.4%)	7 (8.4%)	3 (13%)	0.449
Any history of viral infection events, n (%)		11 (10%)	10 (12%)	1 (4.3%)	0.450
eGFR at vaccination, mL/min/1.73 m^2^		44 (35–54)	43 (35–53)	45 (34–59)	< 0.001

*KT* kidney transplant, *eGFR* estimated glomerular filtration rate.

### Outcomes

**Figure 1:**
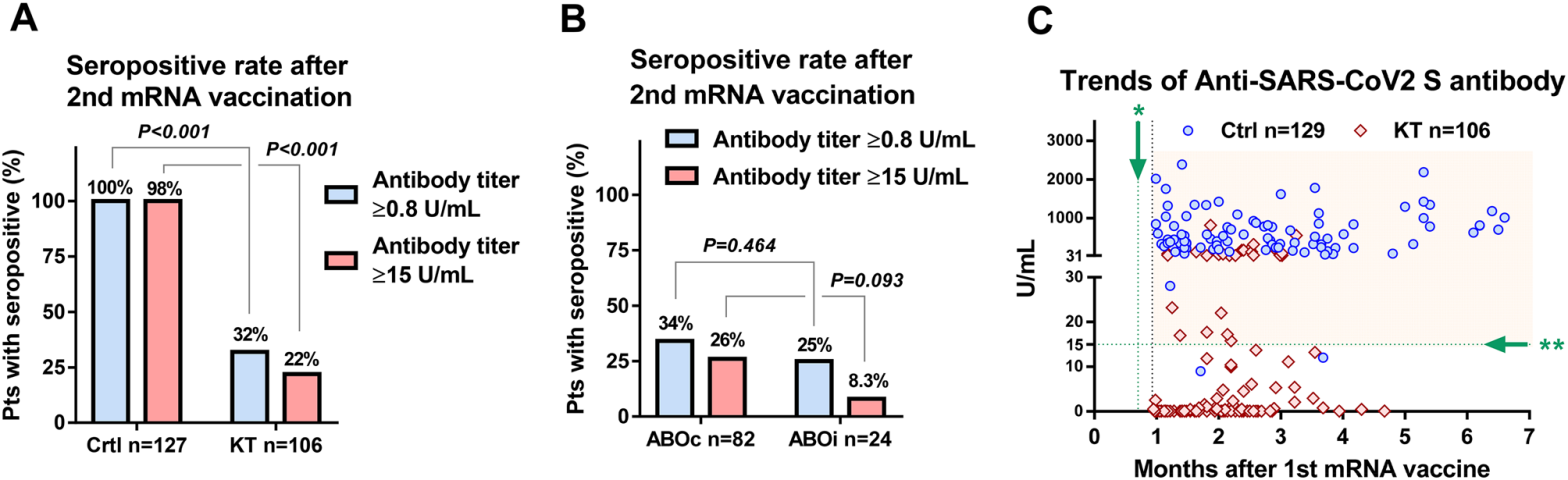
Rate of anti-SARS-CoV-2 S IgG seropositivity after the second dose of the BNT162b2 mRNA vaccine. (**A**) Comparison of seropositivity rates after the second vaccine dose between the control (Ctrl) and kidney transplant (KT) recipients. Seropositivity were defined as anti-SARS-CoV-2 S IgG antibody titers of ≥ 0.80 or ≥ 15 U/mL. (**B**) Comparison of seropositivity rates after the second mRNA vaccine dose between the ABO blood-type compatible (ABOc) and ABO blood-type incompatible (ABOi) KT recipients. (**C**) Trends in anti-SARS-CoV-2 S IgG antibody titers. *Second mRNA vaccination; **cutoff for the presence of neutralizing antibody (≥ 15 U/mL).

The rate of anti-SARS-CoV-2 S IgG antibody titer ≥ 0.8 U/mL was 100% (n = 127/127) and 32% (n = 34/106) in the controls and KT recipients, respectively (P < 0.001; Fig. 1A). The rate of anti-SARS-CoV-2 S IgG antibody titer ≥ 15 U/mL was significantly lower in the KT recipients (22% n = 23/106) than in the controls (98% n = 125/127, P < 0.001; Fig. 1A). The rate of anti-SARS-CoV-2 S IgG antibody titer ≥ 0.8 U/mL and ≥ 15 U/mL was not significantly different in the ABOc KT recipients (34% and 26%, respectively) and ABOi KT recipients (25% and 8.3% respectively) (Fig. 1B). The cross-sectional antibody titers are shown in Fig. 1C.

**Figure 2:**
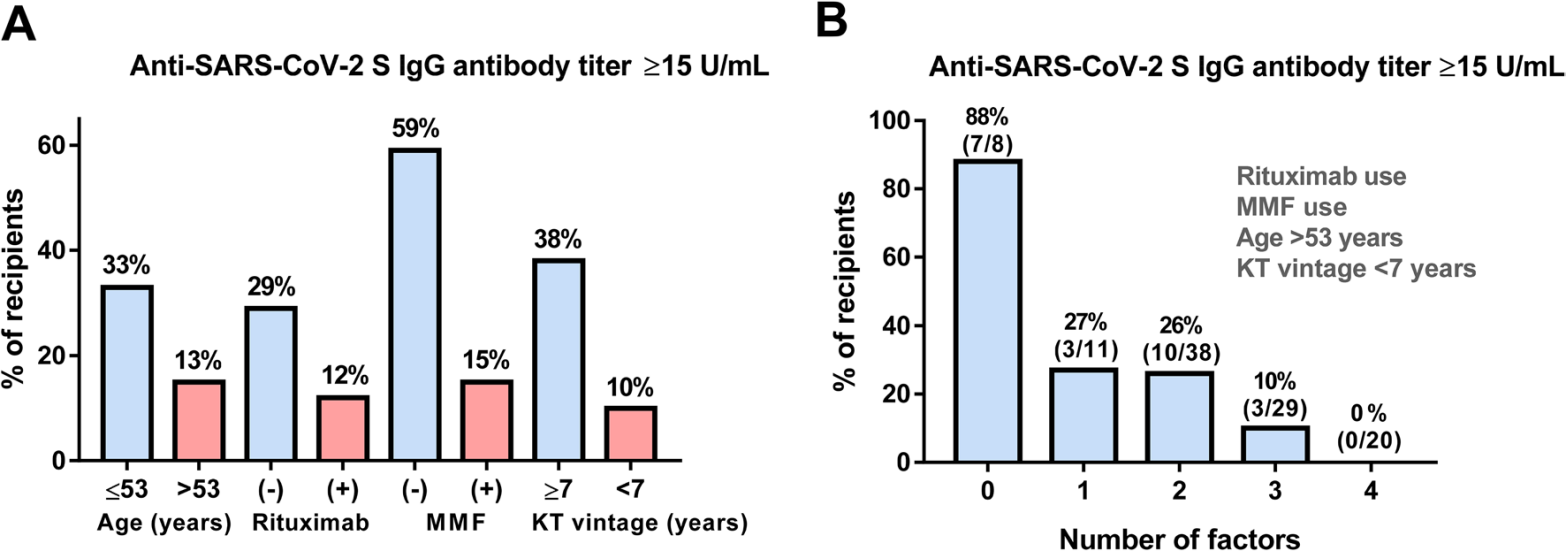
Comparison of rates of anti-SARS-CoV-2 S IgG seropositivity based on select factors. (**A**) Comparison of the anti-SARS-CoV-2 S IgG ≥ 15 U/mL rate based on age (≤ 53 vs. > 53 years), rituximab use (yes vs. no), KT vintage (≥ 7 vs. < 7 years). (**B**) Association between the number of risk factors and the rate of anti-SARS-CoV-2 S IgG ≥ 15 U/mL.

Univariable logistic regression analysis revealed that age (OR 0.94, 95% CI 0.91–0.98, P = 0.004), rituximab use (OR 0.33, 95% CI 0.11–0.97, P = 0.044), MMF use (OR 0.12, 95% CI 0.04–0.37, P < 0.001), and KT vintage (OR 1.10, 95% CI 1.03–1.17, P = 0.005) were significantly associated with anti-SARS-CoV-2 S IgG antibody titer ≥ 15 U/mL in KT recipients (Table 2). Based on the optimal cutoff values for age (53 years) and KT vintage (7 years) using the area under the ROC curve (AUC) analysis, the rates of anti-SARS-CoV-2 S IgG antibody titer ≥ 15 U/mL ranged between 10 and 15% among the KT recipients > 53 years of age, those with a KT vintage of < 7 years, and those who received rituximab or MMF (Fig. 2A). The rates of anti-SARS-CoV-2 S IgG antibody titer ≥ 15 U/mL were higher in those with more than one risk factor. Specifically, the anti-SARS-CoV-2 S IgG antibody titer ≥ 15 U/mL rates of KT recipients harboring 0, 1, 2, 3, and 4 factors were 88%, 27%, 26%, 10%, and 0%, respectively (Fig. 2B). The AUC for the predictive accuracy of anti-SARS-CoV-2 S IgG antibody titer ≥ 15 U/mL was 0.79 in the model including rituximab use, MMF use, age > 53 years, and KT vintage < 7 years. Summary of the present study was shown in the visual abstract as a Supplemental File.

**Table 2:** Univariable logistic regression analysis.

Variable	Factors	P value	OR	95% CI
Age at vaccination	Continuous	0.004	0.94	0.91–0.98
Gender	Male	0.669	0.82	0.32–2.08
Diabetes	Yes	0.493	0.63	0.17–2.38
Type of KT	ABOi	0.088	0.26	0.06–1.22
Immunosuppression	Rituximab use	0.044	0.33	0.11–0.97
	MMF use	< 0.001	0.12	0.04–0.37
	Everolimus use	0.306	1.97	0.54–7.25
	Cyclosporine use	0.306	1.97	0.54–7.25
	Steroids use	0.968	0.97	0.19–5.01
	3 or more agents use	0.177	0.34	0.07–1.63
Biopsy proven rejection events	Yes	0.507	1.63	0.39–6.87
Any viral infections	Yes	0.306	0.33	0.04–2.74
Renal function (eGFR)	mL/min/1.73 m^2^	0.661	1.01	0.97–1.04
KT vintage, years	Continuous	0.005	1.10	1.03–1.17

*OR* odds ratio, *CI* confidence interval.

## Discussion

In the present retrospective study evaluation humoral response after the second BNT162b2 mRNA vaccination in KT recipients in Japan, we found that only 24% of the KT recipients had anti-SARS-CoV-2 IgG antibody titers of ≥ 15 U/mL, while it was 98% in healthy controls. This finding is comparable to previous studies reporting impaired humoral response in KT recipients^3,7^. However, the rate of anti-SARS-CoV-2 IgG seropositivity varies across the studies, ranging from 4.1 to 40.3%, due to differences in study population, sample size and measurement methods. Korh et al. and Danthu et al. reported anti-SARS-CoV-2 IgG seroprevalence rates of 22% (5/23) and 4.1% (3/74), respectively, in KT recipients, based on the LIAISON® SARS-CoV-2 Trimetric-S IgG assay (Diasorin, Italy) with a positive cutoff value of 13.0 arbitrary units/mL^3,7^. Kolb et al. reported a seroprevalence of 37% (10/28) using the Anti-SARS-CoV-2 QuantiVac ELISA for spike protein (Euroimmun, Germany) with a positive cutoff value of 35.2 binding antibody units/mL^6^. Benomane et al. and Bertrand et al. reported seroprevalence rates of 40.3% (64/159) and 17.8% (8/45 recipients), respectively, using the ARCHITECT^®^ IgG II Quant test for spike protein (Abbott Laboratories) with a positive cutoff value of 50.0 arbitrary units/mL^2,5^. Although our observation of impaired humoral response in KT recipients (22%) is consistent with previous studies, the wide range of antibody tests and different cutoffs employed across the studies may contribute to the different results reported by the studies. One study investigated the agreement of three serological tests from Abbott, Roche, and Diasorin^14^ and found a good agreement among the three tests (Cohen’s kappa, 0.71–0.87). However, that study also reported that the clinical performance of these tests was insufficient in studies with low seroprevalence^14^. As there are various antibody assays and they had their cutoffs, the lack of standardization among them may contribute to the different results reported by the studies^15^. Accordingly, The U.S. Food and Drug Administration (FDA) states that “antibody testing is not currently recommended to assess immunity after COVID-19 vaccination” because a result from currently authorized SARS-CoV-2 antibody tests is not an indication of a specific level of immunity or protection from SARS-CoV-2 infection after the person has received a COVID-19 vaccination^16^. Although a potential association between neutralization titers and protection from SARS-CoV-2 infection is reported^17^, 15 U/mL may not be an entirely protective level post-vaccination in the context of breakthrough infection^18^ and omicron variants^19^. Also, the efficacy of SARS-CoV-2 vaccines cannot be measured by IgG antibody titers alone^20^. Overall, these findings highlight the need for careful interpretation of the results from different tests, especially in KT recipients. As the determination of protective IgG antibody levels remains unclear, further studies are necessary for optimal methods and cutoff values to determine the efficacy of SARS-CoV-2 mRNA vaccines.

The current study findings suggest that humoral response after SARS-CoV-2 mRNA vaccination is strongly inhibited in KT recipients > 53 years of age, those treated with rituximab or MMF, and those with a KT vintage of < 7 years. Interestingly, similar factors were reported to be associated with weaker humoral response in a recent study that investigated the SARS-CoV-2 S IgG antibody in 142 KT recipients using the LIAISON^®^ assay and showed age ≥ 54 years, KT vintage ≤ 8 years, and treatment with ≥ 2 immunosuppressants were significantly associated with seroconversion^21^. These results suggest that combination immunosuppressive therapy may induce strong immunosuppression which might interfere with antiviral antibody production for 7–8 years. Factors that impact antibody response in KT recipients should be further investigated.

Among the immunosuppressive agents, rituximab and MMF exhibited a significant impact on humoral response in not only KT recipients but also patients with other chronic clinical conditions. Several studies reported the negative effects of rituximab and MMF use on anti-SARS-CoV-2 IgG seropositivity in KT recipients^22,23^ and in patients with autoimmune inflammatory rheumatic diseases^24^. Kantauskaite et al. showed that MMF-free immunosuppressive regimens were significantly associated with seroconversion (OR 13.25, 95% CI 3.22–54.6, P < 0.001)^22^. The authors suggested that MMF had a dose-dependent unfavorable effect on antibody titers, such as MMF levels of > 1000 mg/day. We also examined the association between anti-SARS-CoV-2 S IgG antibody titer and MMF dose and found that the humoral response was lower in those treated with MMF doses > 1000 mg (6.3%) compared to those who were not treated with MMF (59%) and those treated with 500–1000 mg MMF (16%), which indicated that MMF dose modification might improve immune response to the SARS-CoV-2 vaccine. However, further investigation is warranted to address the balance between rejection and immune acquisition.

Rituximab use was also significantly associated with impaired humoral response in the current study. A multicenter observational study examining SARS-CoV-2 seropositivity in adult patients with autoimmune inflammatory rheumatic diseases (n = 686) reported that rituximab use was significantly associated with impaired humoral response to the BNT162b2 mRNA vaccine^24^. As B cell depletion is associated with a lack of serological response, those findings are reasonable regarding the negative impact of rituximab on humoral response to various vaccines^25^. Therefore, these results emphasize the importance of SARS-CoV-2 vaccination before the administration of MMF and/or rituximab in KT recipients.

The impact of ABOi KT on humoral response to SARS-CoV-2 vaccines should be addressed. Albeit uncommon across the globe, ABOi KT is a common alternative for donor exchange programs in Japan. As it requires extensive immunosuppression including rituximab and therapeutic apheresis, we hypothesized that ABOi KT might also have a great impact on anti-SARS-CoV-2 S IgG seropositivity. However, we found that ABOi KT had a limited impact on seropositivity in the present study; this result might be associated with the lower statistical power due to the limited sample size, which should be addressed in future studies.

The major limitations of the present study include the limited sample size and retrospective study design. The antibody titer decline over time may affect the result interpretation of this study because the measurement periods are not aligned. The IgG antibody titers were determined during the early phase of mass immunization in Japan. ABOi KT and rituximab use in immunologically high-risk recipients might not be common worldwide. Measurement of antibody titers is one of the several methods to assess immunologic response to vaccination. We assessed on a single platform of antibody measurement in this study. Furthermore, an antibody titer of ≥ 15 U/mL may not be an entirely protective level post-vaccination. Despite those limitations, this is the first study evaluated the seroprevalence of SARS-CoV-2 S IgG antibodies after the second BNT162b2 mRNA vaccine in Japanese KT recipients.

In conclusion, we confirmed that the rate of anti-SARS-CoV-2 IgG seroconversion was low in KT recipients after the second BNT162b2 mRNA vaccine. However, several outstanding questions remain and further investigation is warranted to determine the duration of immunity under immunosuppressive therapy, the effect of reduced titers on the protective activity of vaccines against breakthrough infections, and the efficacy of third vaccination in KT recipients.

## Methods

This retrospective study was approved by the Ethics Committee of Hirosaki University (2021-089). All participants had previously provided written informed consent for other biomarker studies. Additional informed consent for the current study was waived with approval by the Ethics Committee of Hirosaki University. The clinical and research activities being reported are consistent with the Principles of the Declaration of Istanbul and Helsinki.

### Participants

The current study conducted between June 21, 2021 and November 1, 2021 included 106 KT recipients and 127 healthy controls who received the second BNT162b2 dose at least 7 days before the measurement of anti-SARS-CoV-2 antibody titers. The control group included members of the medical staff, medical students, and posttreatment patients with localized cancers who were not actively receiving any treatment. Those with previous SARS-CoV-2 infection and who provided blood samples for titer measurement within the first 7 days after the second BNT162b2 dose were excluded. Clinical parameters of age, gender, primary kidney disease, KT vintage (years), dialysis vintage (years), ABO blood type compatibility, immunosuppressant agents, past history of rejection events, past history of viral events, and renal function were obtained from the medical records.

### Immunosuppression

Flow cytometry and Luminex-based single-antigen bead assay were used to select the immunosuppression protocol^26^. Low-dose rituximab (100 mg/m^2^ or 100 mg/body) and donor-specific human leukocyte antigen antibodies were administered in recipients of ABOi and ABO blood-type compatible (ABOc) KT, respectively. For ABOc KT recipients, basic immunosuppression included calcineurin inhibitors (CNIs), mycophenolate mofetil (MMF), and steroids. Most KT recipients, i.e., those who underwent KT after February 2002, were treated with induction therapy using the anti-CD25 monoclonal antibody basiliximab on the day of operation and postoperative day 4. Intravenous immunoglobulin was not given in all KT patients due to the lack of insurance coverage ABOi KT recipients received basic immunosuppressive agents, rituximab and therapeutic apheresis. Low-dose rituximab was administered three weeks before transplantation. Basic immunosuppressive agents (CNIs, MMF, and steroids) were administered 7 days before transplantation in all ABOi KT recipients. Several sessions of double-filtration plasmapheresis and one session of plasma exchange were performed on the day before surgery to remove anti-A/B antibodies until the anti-A/B antibody titers decreased to a level of < 1:32–1:64. Those with viral infection or malignancies were switched from CNIs or MMF to everolimus.

### Measurement of anti-SARS-CoV-2 IgG antibody titers

We measured IgG antibodies against the SARS-CoV-2 spike (S) protein receptor-binding domain. Cross-sectional blood samples collected for regular evaluation were used. Anti-SARS-CoV-2 S IgG antibody titer was quantitatively detected with a double-antigen sandwich-based electrochemiluminescence immunoassay (ECLIA), using the Elecsys^®^ Anti-SARS-CoV-2 S RUO (Covas 8000/e 801; Roche Diagnostics, Mélan, France). According to the manufacturer’s data, the measurement range of this assay is between 0.40 and 250 U/mL, and values above 0.80 U/mL are considered positive. The specificity of this assay is 99.98% and the sensitivity is 98.8%. We defined seropositivity as an anti-SARS-CoV-2 S IgG level of ≥ 0.80 U/mL or ≥ 15 U/mL. anti-SARS-CoV-2 S IgG level ≥ 15 U/mL was shown to be sufficient for the presence of neutralizing antibodies with the positive percent agreement: 88.87%, negative predictive value: 90.00%, and positive predictive value: 99.10%.

### Outcomes

In the current study, we compared the rates of anti-SARS-CoV-2 S IgG seroprevalence (anti-SARS-CoV-2 S IgG level of ≥ 0.8 U/mL or ≥ 15 U/mL) between the controls and KT recipients. We also compared the anti-SARS-CoV-2 IgG seroprevalence rate in ABOi KT recipients, and investigated the factors associated with anti-SARS-CoV-2 IgG S IgG antibody titer ≥ 15 U/mL after the second SARS-CoV-2 mRNA vaccination in KT recipients.

### Statistical analysis

Qualitative and quantitative variables were described as numbers with percentages and medians with interquartile ranges (IQRs), respectively. The chi-squared, Fisher’s exact, Mann–Whitney *U*, and Student’s t tests were used for the statistical comparison between the healthy controls and KT recipients. Univariable logistic regression analysis was performed to identify factors associated with anti-SARS-CoV-2 IgG seropositivity after the second SARS-CoV-2 mRNA vaccination, and odds ratio (OR) with 95% confidence interval (CI) were calculated. Predictive accuracy and optimal cutoff values for anti-SARS-CoV-2 IgG levels were evaluated by area under the receiver operating characteristic (ROC) curve analysis. All statistical analyses were performed using BellCurve for Excel 3.10 (Social Survey Research Information, Tokyo, Japan) and GraphPad Prism 7.00 (GraphPad Software, San Diego, CA, USA).

## Supplementary Information

Supplementary Information 1.

Supplementary Information 2.

## Data Availability

Our data can be shared on reasonable request: Email: shingoh@hirosaki-u.ac.jp.
