# Supplementary material for: Seroprevalence of SARS-CoV-2 spike IgG antibodies after the second BNT162b2 mRNA vaccine in Japanese kidney transplant recipients
Source: Sci Rep. 2022 Apr 7;12:5876. doi: 10.1038/s41598-022-09897-0 (PMC8988536; doi:10.1038/s41598-022-09897-0)
Supplement: Supplementary file 1 — Supplementary Information 1. [file 41598_2022_9897_MOESM1_ESM.docx]

**The supplemental figure: visual abstract**

Summary and key findings of the present study. The seroprevalence rate of the healthy controls and kidney transplant recipients is 98% and 22%, respectively. **Seroprevalence was not significantly associated with ABO blood type incompatibility.** Humoral response after the second BNT162b2 mRNA vaccine is greatly hindered by immunosuppression therapy in kidney transplant recipients. Older age, rituximab use, mycophenolate mofetil use, and kidney transplant vintage may play key roles in seroconversion.
