# Supplementary material for: Seroprevalence of SARS-CoV-2 spike IgG antibodies after the second BNT162b2 mRNA vaccine in Japanese kidney transplant recipients
Source: Sci Rep. 2022 Apr 7;12:5876. doi: 10.1038/s41598-022-09897-0 (PMC8988536; doi:10.1038/s41598-022-09897-0)
Supplement: Supplementary file 2 — Supplementary Information 2. [file 41598_2022_9897_MOESM2_ESM.pdf]

Seroprevalence of SARS-CoV-2 spike IgG antibodies after the second BNT162b2 mRNA vaccine in Japanese kidney transplant recipients

Methods

The BNT162b2 mRNA vaccine, two doses, 21 days interval

Controls n=127

Kidney transplant (KT) recipients n=106

ABO compatible (ABOc) KT recipients n=82

ABO incompatible (ABOi) KT recipients n=24

The rate of anti–SARS-CoV-2 IgG seropositivity after the second SARS-CoV-2 mRNA vaccination

Outcomes

Hamaya T. et al

Anti-SARS-CoV-2 S IgG

antibody titer ≥15 U/mL

Controls **98%** vs. KT recipients **22%**

ABO compatible **26%** vs. ABO incompatible **8.3%**

Factors associated with impaired humoral response

1. Age >53 years
2. KT vintage <7 years
3. Rituximab use
4. Mycophenolate mofetil use

Conclusion: Humoral response after the second BNT162b2 mRNA vaccine was greatly hindered by immunosuppression therapy in KT recipients.

The Supplemental file
